# Microbial dysbiosis and fecal metabolomic perturbations in Yorkshire Terriers with chronic enteropathy

**DOI:** 10.1038/s41598-022-17244-6

**Published:** 2022-07-28

**Authors:** Alexandra I. Galler, Jan S. Suchodolski, Joerg M. Steiner, Chi-Hsuan Sung, Katharina M. Hittmair, Barbara Richter, Iwan A. Burgener

**Affiliations:** 1 Small Animal Internal Medicine, University of Veterinary Medicine, Vienna, Austria; 2 Gastrointestinal Laboratory, Department of Small Animal Clinical Sciences, Texas A&M University, College Station, TX USA; 3 Clinical Unit of Diagnostic Imaging, University of Veterinary Medicine, Vienna, Austria; 4 Institute of Pathology, University of Veterinary Medicine, Vienna, Austria

**Keywords:** Diseases, Gastroenterology

## Abstract

Dysbiosis and perturbations of fecal metabolic profiles have been reported in dogs with inflammatory bowel disease. Currently the incidence of dysbiosis and the fecal metabolomic profile in Yorkshire Terriers with chronic enteropathy (YTE) and the effects of treatment are unknown. This prospective observational study analyzed the dysbiosis index (DI) and fecal bile acid, sterol and fatty acid profiles in 14 Yorkshire Terriers with active YTE, 11 dogs in clinical remission, and 26 healthy Yorkshire Terriers. YTE was associated with dysbiosis and a significant increase in fatty acids (docosanoate, *p* = 0.002; gondoate, *p* = 0.026; erucate, *p* < 0.001; nervonate, *p* < 0.001; linolenate, *p* < 0.001), and plant sterols (campesterol, *p* < 0.001; brassicasterol, *p* = 0.024). The abundances of *Fusobacterium (p* < 0.001) and *Cl. hiranonis (p* = 0.018) and the concentrations of the secondary bile acid ursodeoxycholic acid (*p* = 0.033) and the plant sterol sitostanol (*p* = 0.003) were significantly decreased compared to healthy dogs. Dysbiosis, abundances of *Fusobacterium, Cl. hiranonis* and fecal concentrations of bile acids and sterols did not recover after treatment, while fecal fatty acid concentrations decreased in treated dogs. YTE is associated with dysbiosis and changes in bile acid, fatty acid, and sterol metabolism. These changes only recovered partially despite clinical remission. They might be breed-specific and involved in the pathogenesis of YTE.

## Introduction

Canine inflammatory bowel disease (IBD) represents a group of enteropathies characterized by chronic signs of gastrointestinal disease, exclusion of systemic, infectious, endocrine, and neoplastic causes, as well as histopathologic evidence of intestinal mucosal inflammation^1,2^. The etiology of the disease is not well understood, although it is considered to result from a complex interplay between the genetic background, environmental factors, the intestinal immune system, and the intestinal microbiome^3–6^. A wide array of organisms colonize the gut and the interaction between these intestinal microbes and their host is critical in health and disease. The intestinal microbiome protects against pathogens, educates the immune system, and has important metabolic functions^7^. The dysbiosis index (DI) is a PCR-based assay that was developed to quantify intestinal dysbiosis in dogs. It assesses the abundance of total bacteria as well as the abundances of seven selected bacterial groups with important metabolic functions and combines them into a single numeric value^8^. The metabolome encompasses all small molecules present in a biological system and comprises exogenous, endogenous, and gut microbially-derived metabolites, such as carbohydrates, amino acids, lipids, bile acids, and their modified products^9^. Metabolomic analyses could potentially address key issues of IBD and could provide valuable tools in clinical diagnosis and assessment of treatment response. Intestinal dysbiosis and changes in fecal metabolomic profiles, especially regarding short-chain fatty acid and bile acid metabolism have been reported in dogs with IBD; however, data are scarce and inconsistent^10–13^.

Different definitions for IBD and confounding factors such as previous treatment, age or sex, and different methodological approaches may have caused discrepancies between previous studies. In addition, conflicting results may be caused by the multifactorial nature of IBD and the wide variation in phenotype and severity. IBD is likely to be a syndrome that encompasses several disease subtypes. Although IBD or chronic enteropathy (CE) can affect any dog, some breeds display breed-specific disease phenotypes^14–16^. In the Yorkshire Terrier, retrospective studies have described a protein losing enteropathy syndrome that is distinct from other breeds^14,15^, suggesting the existence of a breed-specific "Yorkshire Terrier enteropathy" (YTE). Affected Yorkshire Terriers may show clinical signs associated with intestinal protein loss and low oncotic pressure^14,15^. Histopathologic abnormalities include severe intestinal lymphatic dilation, crypt dilatation and crypt abscesses, villous stunting, and inflammatory infiltrates of the lamina propria of variable severity^15^. The fecal microbiome and metabolomic profiles in YTE have not been investigated so far and may differ from CE dogs of various other breeds. Furthermore, limited information is available concerning the influence of different treatments on intestinal dysbiosis and the fecal metabolome in dogs with CE.

The purpose of this study was to investigate the DI and the fecal metabolome of treatment naïve Yorkshire Terriers with YTE and comparison to healthy Yorkshire Terriers. Furthermore, we studied the impact of treatment on dysbiosis and metabolomic profiles. We hypothesized that YTE would be associated with changes in DI and fecal metabolomic profiles and that these changes would recover after successful treatment.

## Results

### Animal characteristics

#### YTE with active disease

The YTE group (n = 14) included 10 female (5 spayed) and 4 male (2 neutered) Yorkshire Terriers. The mean age was 5.9 years (+/−1.7 years), and mean body weight was 3.5 kg (+/−1.4 kg). At the time of presentation, dogs were fed various commercial diets (12 canned and 2 dry foods). Eleven dogs were presented with a history of chronic or intermittent gastrointestinal signs and 3 dogs because of abdominal effusion. The dogs were diagnosed with mild to very severe YTE (median canine chronic enteropathy activity index (CCECAI) score 10.5, range 5–13).

Endoscopic duodenal scores ranged from 0 to 3 with a median score of 2 (maximal possible score 4). Histological duodenal World Small Animal Veterinary Association (WSAVA) scores indicated mild or moderate histological changes (median 4.5, range 2–11). In all dogs a predominantly lymphoplasmacytic infiltration of the intestinal mucosa was found. Seven dogs showed an additional eosinophilic and four dogs an additional neutrophilic infiltration. Other histological abnormalities were villus blunting in 4/11 dogs where villus length could be evaluated, increased intraepithelial lymphocytes (3/14), and fibrosis of the propria (3/14). Furthermore, crypt lesions and lymphangiectasia were present in 4 and 3 dogs, respectively.

#### YTE in clinical remission

Eleven dogs were available for re-evaluation after achieving clinical remission. Nine dogs were clinically well controlled receiving a hydrolyzed diet. In 8 of them the hydrolyzed diet was the first diet that was introduced. One dog was switched from a low fat to a hydrolyzed diet before achieving clinical remission. Fecal sampling of the dogs was repeated 69–119 days (median 70 days) post diagnosis. One dog was clinically well controlled receiving a low fat diet and was sampled after 70 days. One dog achieved clinical remission after the administration of prednisolone and was sampled again 126 days post diagnosis (71 days after initiation of prednisolone). One dog did not finish the study due to poor owner compliance. Two dogs had not finished the treatment trial at the time of sample analysis. The CCECAI scores had decreased in all re-evaluated dogs (n = 11, median 2, range 1–3).

#### Healthy control group

The control group (n = 26) consisted of 14 female (8 spayed) and 12 (6 neutered) male Yorkshire Terriers. The mean age of the dogs was 8.2 years (+/−3.2 years). The mean body weight was 3.9 kg (+/−1.35). Twenty-five dogs in the control group received various commercial diets (19 canned and 6 dry foods). One dog was fed with a Bones and Raw Food (BARF) diet. Sex and body weight did not differ significantly between healthy and YTE dogs. Dogs in the healthy control group were older than dogs with YTE (*p* = 0.04). The median CECCAI score in the healthy control group was 0.5 (range 0–3), which was significantly lower compared to dogs with YTE (*p* < 0.001).

### Dysbiosis index and fecal metabolomics in YTE dogs compared to healthy control dogs

#### Dysbiosis Index

Seven YTE dogs had DI values > 2, indicating a shift in the intestinal microbiome. In 1 dog the DI was mildly increased (0–2), suggesting a minor shift in the microbiota. Six YTE dogs had a negative DI, but in 5 of these dogs the abundance of individual bacteria was outside the respective reference interval, indicating minors forms of dysbiosis in those dogs as well. In all of these 5 dogs the abundance of *Faecalibacterium* was decreased, either as a single change or in combination with other altered groups. The abundance of *Clostridium hiranonis (Cl. hiranonis)* was decreased in 7 dogs with YTE and that of *Faecalibacterium* was decreased in 8 dogs with YTE. Of the healthy control dogs 4 had a DI > 2 and 4 had values between 0 and 2, suggesting that several of the control dogs also had shifts in the intestinal microbiome. Eighteen healthy control dogs had negative DI values, with 3 of them having an abundance of one bacterial group outside the respective reference interval, indicating a minor form of dysbiosis.

**Figure 1:**
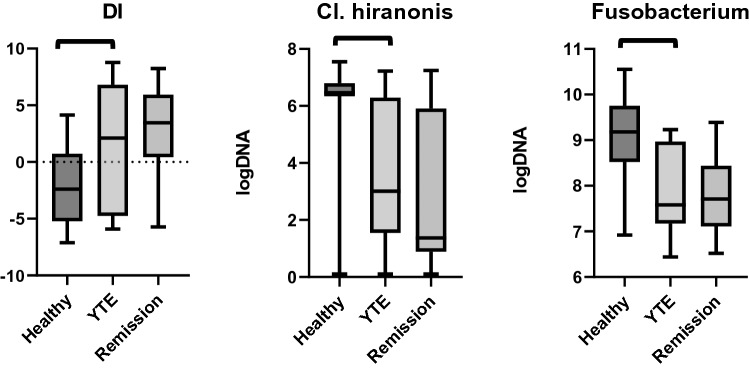
Box plots comparing the DI and the abundances of *Cl. hiranonis and Fusobacterium* in healthy controls, dogs with YTE, and dogs with YTE after achieving clinical remission. Square brackets are indicating significant differences between groups. Box plots represent the 25–75th percentile; the median is shown as the heavy dark horizontal line. Vertical lines extend to the minimum and maximum values. DI, dysbiosis index; YTE, Yorkshire Terrier enteropathy.

The DI was significantly higher in dogs with YTE compared to healthy control dogs (*p* = 0.048). The abundances of *Fusobacterium* (*p* < 0.001) and *Cl. hiranonis* (*p* = 0.018) were significantly lower in dogs with YTE compared to healthy control dogs (Table 1; Fig. 1). The DI correlated positively with CCECAI scores in YTE dogs (ρ = 0.55, *p* = 0.042).

**Table 1:** Summary statistics of the abundances of bacterial groups assessed for the calculation of the DI in healthy and YTE dogs.

		Reference	Mean	SD	Median	Minimum	Maximum	*P* value
Turicibacter Log DNA	Healthy	4.6–8.1	6.42	1.11	6.17	4.03	8.70	0.664
	YTE		6.53	1.34	6.46	4.33	8.41	
E.coli Log DNA	Healthy	0.9–8.0	4.79	2.00	4.97	1.39	7.82	0.214
	YTE		5.84	2.55	6.81	1.39	9.18	
Fusobacterium Log DNA	Healthy	7.0–10.3	9.10	0.92	9.24	6.92	10.55	< 0.001
	YTE		7.87	0.95	7.58	6.44	9.23	
Faecalibacterium Log DNA	Healthy	3.4–8.0	4.26	1.63	3.71	1.08	7.12	0.130
	YTE		3.47	1.15	3.26	2.26	6.34	
Blautia Log DNA	Healthy	9.5–11.0	10.40	0.54	10.47	9.16	11.22	0.967
	YTE		10.34	0.67	10.61	8.67	10.90	
Cl. hiranonis Log DNA	Healthy	5.1–7.1	5.90	2.19	6.48	0.10	7.55	0.018
	YTE		3.89	2.65	4.23	0.10	7.22	
Streptococcus Log DNA	Healthy	1.9–8.0	4.95	1.49	4.51	2.83	8.81	0.410
	YTE		5.65	2.26	5.07	2.28	8.91	
Dysbiosis Index	Healthy		− 2.34	3.37	− 2.48	− 7.11	3.82	0.048
	YTE		1.32	5.45	2.11	-5.90	8.77	

DI, dysbiosis index, YTE, Yorkshire Terrier enteropathy; SD, standard deviation.

#### Fecal bile acid concentrations

**Figure 2:**
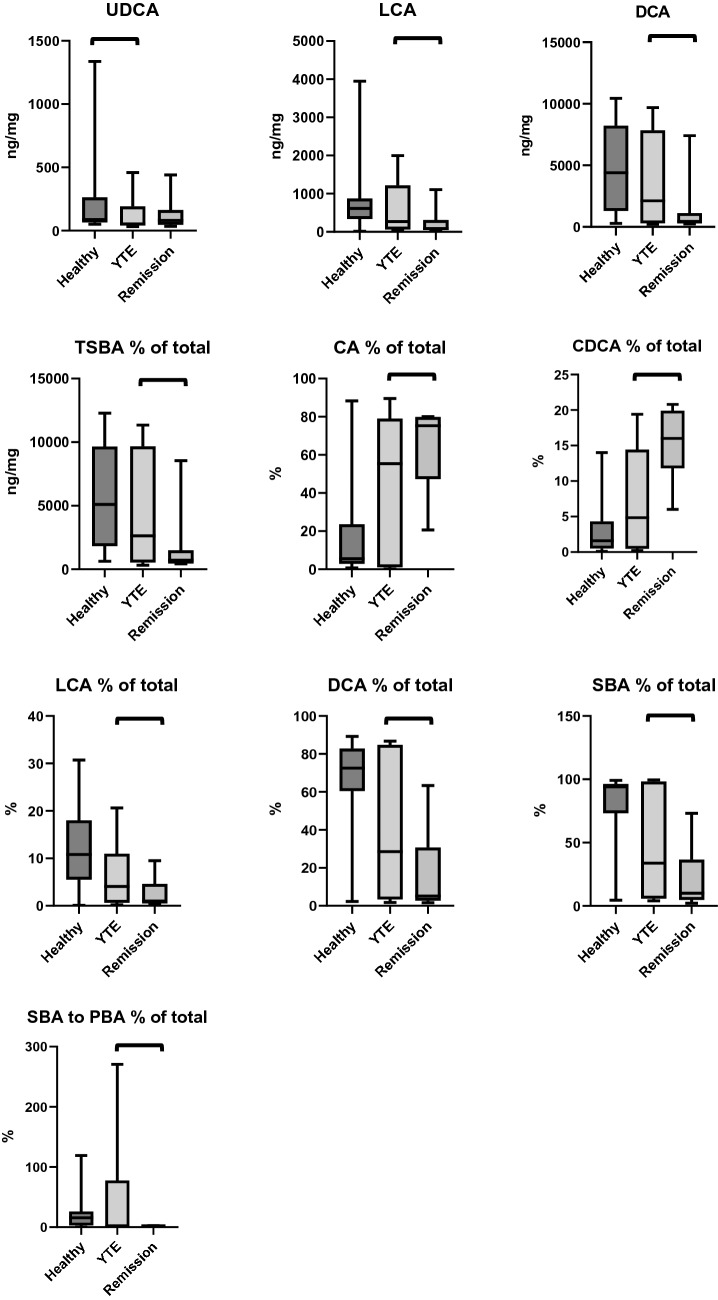
Box plots comparing total amounts and percentages of fecal bile acid concentrations and the ratio of secondary to primary fecal bile acids in healthy controls, dogs with YTE, and dogs with YTE after achieving clinical remission. Square brackets are indicating significant differences between groups. Box plots represent the 25–75th percentile; the median is shown as the heavy dark horizontal line. Vertical lines extend to the minimum and maximum values. UDCA, ursodeoxycholic acid; LCA, lithocholic acid; CDA, chenodeoxycholic acid; DCA, deoxycholic acid; TSBA, total secondary bile acids; CA, cholic acid; CDCA, chenodeoxycholic acid; SBA, secondary bile acids; SBA to PBA, ratio of primary to secondary bile acids; YTE, Yorkshire Terrier enteropathy.

The concentration of ursodeoxycholic acid (UDCA), a secondary bile acid, was significantly lower in the feces of YTE dogs compared to healthy control dogs (*p* = 0.033) (Table 2; Fig. 2).

**Table 2:** Summary statistics of fecal bile acid concentrations in healthy and YTE dogs.

		Mean	SD	Median	Minimum	Maximum	P value
CA (ng/mg)	Healthy	2476	4479	285	62	16,757	0.620
	YTE	4231	5517	908	14	16,358	
CDA (ng/mg)	Control	310	605	82	12	2897	0.150
	YTE	606	899	285	21	3312	
LCA (ng/mg)	Control	780	798	613	14	3949	0.650
	YTE	628	707	269	28	1998	
DCA (ng/mg)	Control	4784	3636	4401	281	10,438	0.230
	YTE	3993	4018	2122	222	9694	
UDCA (ng/mg)	Control	217	290	88	50	1337	0.033
	YTE	122	130	53	31	460	
TPBA (ng/mg)	Control	2786	5043	442	77	19,654	0.530
	YTE	4872	6372	1193	36	19,670	
TSBA (ng/mg)	Control	5781	4065	5102	629	12,277	0.190
	YTE	4743	4623	2636	313	11,347	
TBA (ng/mg)	Control	8567	4739	8891	1461	20,676	0.520
	YTE	9615	5089	9726	1616	20,783	
SBA (%)	Control	76	32	94	5	99	0.590
	YTE	50	44	34	4	100	
PBA (% )	Control	24	32	6	1	95	0.950
	YTE	50	44	66	0	96	
CA (%)	Control	21	29	5	1	88	0.700
	YTE	42	38	55	0	89	
CDA (%)	Control	3	3	2	0	14	0.210
	YTE	7	7	5	0	19	
LCA (%)	Control	12	8	11	0	31	0.110
	YTE	7	7	4	0	21	
DCA (%)	Control	62	28	72	2	89	0.480
	YTE	42	38	29	2	87	
UDCA (%)	Control	3	4	2	0	18	0.700
	YTE	1	1	1	0	2	
SBA to PBA	Control	22	29	16	0	119	0.620
	YTE	51	87	1	0	271	
PBA to SBA	Control	2	6	0	0	21	0.360
	YTE	7	9	2	0	23	

CA, cholic acid; CDCA, chenodeoxycholic acid; LCA, lithocholic acid; DCA, deoxycholic acid; UDCA, ursodeoxycholic acid; TPBA, total primary bile acids; TSBA, total secondary bile acids; SBA to PBA; ratio of secondary to primary bile acids; PBA to SBA, ratio of primary to secondary bile acids; YTE, Yorkshire Terrier enteropathy; SD, standard deviation.

In YTE dogs, total fecal primary bile acid concentrations and the percentage of primary bile acids correlated negatively with the abundance of *Cl. hiranonis* (ρ  = − 0.70, *p* = 0.006; ρ  = − 0.67, *p* = 0.009) and positively with the DI (ρ  = 0.70, *p* = 0.006; ρ  = 0.63, *p* = 0.016). Concentrations and percentages of total secondary bile acids correlated negatively with the DI (ρ = − 0.60, *p* = 0.022; ρ = − 0.63, *p* = 0.016). Percentages of fecal secondary bile acids correlated positively with abundances of *Cl. hiranonis* (ρ = 0.67, *p* = 0.009). Total fecal bile acid concentrations correlated positively with WSAVA histopathology scores in YTE dogs (ρ = 0.80, *p* < 0.001).

#### Fecal sterol concentrations

**Figure 3:**
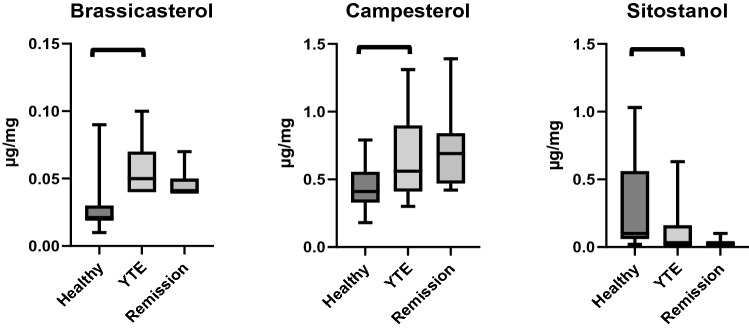
Box plots comparing fecal concentrations of brassicasterol, campesterol, and sitostanol in healthy controls, dogs with YTE, and dogs with YTE after achieving clinical remission. Square brackets are indicating significant differences between groups. Box plots represent the 25–75th percentile; the median is shown as the heavy dark horizontal line. Vertical lines extend to the minimum and maximum values. YTE, Yorkshire Terrier enteropathy.

Fecal concentrations of 2 plant sterols, campesterol (*p* < 0.001) and brassicasterol (*p* = 0.024), were increased in YTE dogs compared to healthy control dogs, while fecal concentrations of sitostanol, a plant sterol metabolite, were lower (*p* < 0.001) in YTE dogs (Table 3; Fig. 3).

**Table 3:** Summary statistics of fecal sterol concentrations in healthy and YTE dogs.

		Mean	SD	Median	Minimum	Maximum	P value
Coprostanol (µg/mg)	Healthy	0.06	0.06	0.04	0.01	0.25	0.851
	YTE	0.05	0.02	0.04	0.02	0.07	
Cholesterol (µg/mg)	Healthy	5.11	3.18	4.50	1.60	14.64	0.740
	YTE	5.26	2.93	4.84	1.73	12.00	
Cholestanol (µg/mg)	Healthy	0.32	0.20	0.25	0.12	0.95	0.067
	YTE	0.24	0.20	0.21	0.11	0.90	
Brassicasterol (µg/mg)	Healthy	0.03	0.02	0.02	0.01	0.09	< 0.001
	YTE	0.05	0.02	0.05	0.04	0.10	
Lathosterol (µg/mg)	Healthy	0.03	0.02	0.03	0.01	0.14	0.942
	YTE	0.04	0.04	0.03	0.01	0.18	
Campesterol (µg/mg)	Healthy	0.44	0.17	0.41	0.18	0.79	0.021
	YTE	0.67	0.31	0.56	0.30	1.31	
Stigmasterol (µg/mg)	Healthy	0.18	0.10	0.16	0.04	0.41	0.942
	YTE	0.17	0.09	0.18	0.06	0.35	
Fusosterol (µg/mg)	Healthy	0.06	0.02	0.06	0.02	0.09	0.093
	YTE	0.05	0.05	0.03	0.02	0.21	
Beta-sitosterol (µg/mg)	Healthy	1.21	0.60	1.26	0.16	2.56	0.239
	YTE	1.02	0.77	0.76	0.27	2.84	
Sitostanol (µg/mg)	Healthy	0.28	0.29	0.10	0.02	1.03	0.003
	YTE	0.11	0.18	0.02	0.01	0.63	
TM phytosterols	Healthy	2.17	1.02	2.22	0.45	4.67	0.690
	YTE	2.02	1.25	1.82	0.70	4.96	
TM zoosterols	Healthy	5.53	3.38	4.86	1.80	15.82	0.740
	YTE	5.59	2.99	5.03	2.05	12.43	
TM phyto- to zoosterols	Healthy	0.55	0.41	0.47	0.08	1.41	0.443
	YTE	0.47	0.41	0.37	0.06	1.48	
TM sterols (µg/mg)	Healthy	7.72	3.53	6.36	4.12	18.27	1.000
	YTE	7.66	3.20	7.69	3.68	13.23	
TM sterols to fatty acids	Healthy	0.33	0.19	0.31	0.10	0.87	0.740
	YTE	0.30	0.16	0.26	0.08	0.72	

TM, total measured; TM Phyto- to Zoosterols; ratio of total measured phyto- to total measured zoosterols; TM Sterols to Fatty Acids; ratio of total measured sterols to total measured fatty acids; YTE, Yorkshire Terrier enteropathy; SD, standard deviation.

In YTE dogs fecal concentrations of cholesterol (ρ = 0.55, *p* = 0.041), lathosterol (ρ = 0.58, *p* = 0,030), and total measured zoosterols (ρ = 0.54, *p* = 0.048) correlated positively with WSAVA histopathology scores. Fecal brassicasterol (ρ = 0.61, *p* = 0.021) and fusosterol (ρ = 0.55, *p* = 0.043) concentrations correlated positively with *Blautia* abundances in YTE dogs. Furthermore, fecal sitostanol concentrations (ρ = 0.66, *p* = 0.010) and total sterol concentrations (ρ = 0.64, *p* = 0.014) correlated positively with the abundance of *Faecalibacterium*.

#### Fecal fatty acid concentrations

**Figure 4:**
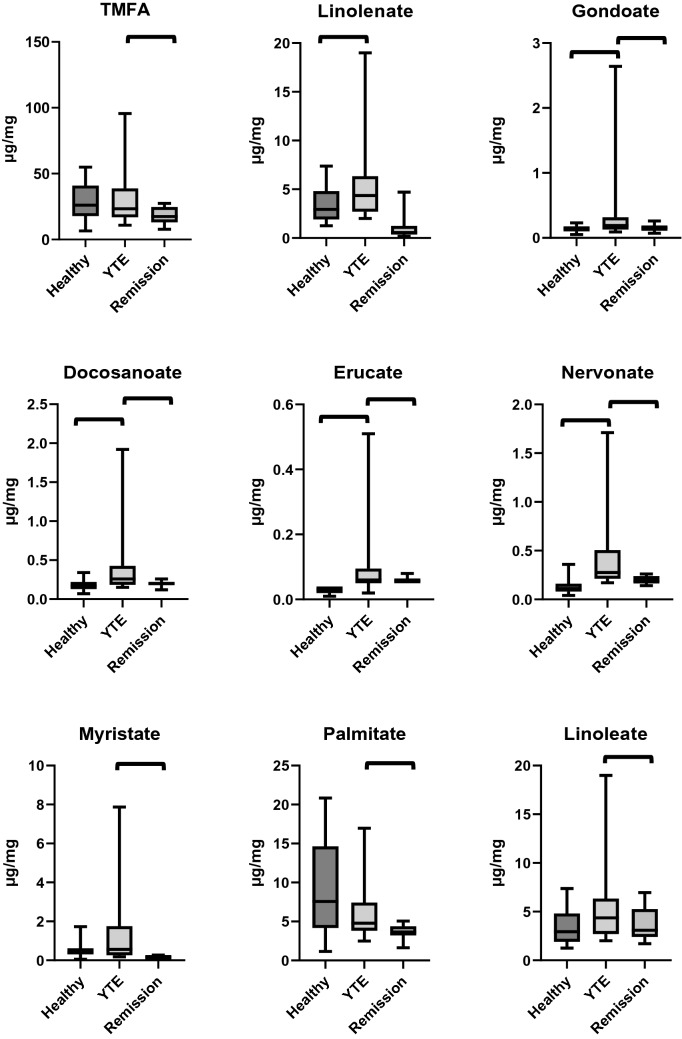

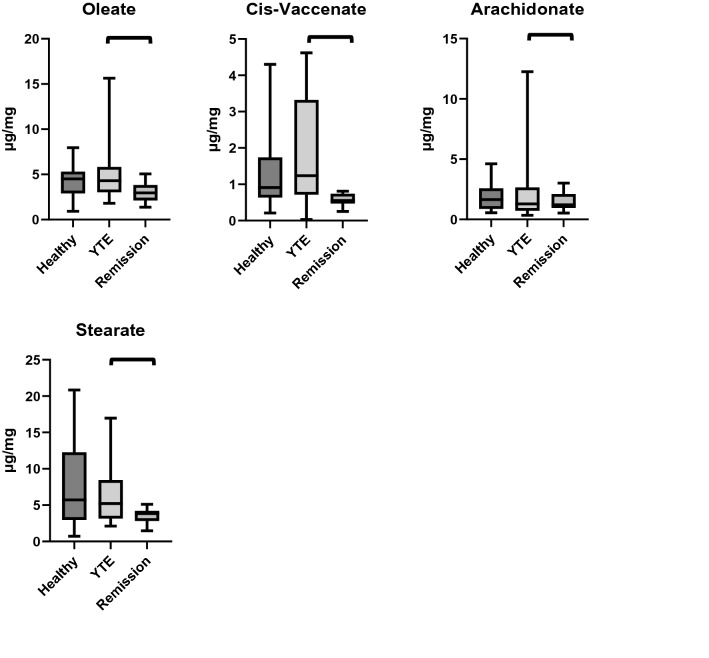
Box plots comparing fecal fatty acid concentrations in healthy controls, dogs with YTE, and dogs with YTE after achieving clinical remission. Square brackets are indicating significant differences between groups. Box plots represent the 25–75th percentile; the median is shown as the heavy dark horizontal line. Vertical lines extend to the minimum and maximum values. TMFA, total measured fatty acids; YTE, Yorkshire Terrier Enteropathy.

Fecal concentrations of the long-chain fatty acids docosanoate (*p* = 0.002), gondoate (*p* = 0.026), erucate (*p* < 0.001), nervonate (*p* < 0.001), and linolenate (*p* < 0.001) were higher in YTE dogs compared to healthy control dogs. (Table 4; Fig. 4)

**Table 4:** Summary statistics of fecal fatty acid concentrations in YTE dogs and healthy control dogs.

		Mean	SD	Median	Minimum	Maximum	P value
Myristate (µg/mg)	Healthy	0.54	0.40	0.40	0.05	1.73	0.263
	YTE	1.39	2.10	0.57	0.18	7.87	
Palmitate (µg/mg)	Healthy	8.97	5.81	7.57	1.16	20.85	0.239
	YTE	6.63	4.34	4.79	2.49	16.97	
Linoleate (µg/mg)	Healthy	3.37	1.79	2.94	1.26	7.37	0.051
	YTE	5.50	4.34	4.36	2.01	18.99	
α-Linolenate (µg/mg)	Healthy	0.25	0.18	0.20	0.04	0.74	< 0.001
	YTE	0.94	0.84	0.73	0.26	3.51	
Oleate (µg/mg)	Healthy	4.20	1.82	4.51	0.92	7.95	0.675
	YTE	5.64	4.32	4.30	1.80	15.64	
Cis-vaccenate (µg/mg)	Healthy	1.45	1.27	0.91	0.21	4.30	0.409
	YTE	1.89	1.44	1.24	0.03	4.62	
Stearate (µg/mg)	Healthy	7.80	6.08	5.72	0.71	20.85	0.718
	YTE	6.58	4.71	5.21	2.12	16.97	
Arachidonate (µg/mg)	Healthy	1.90	1.15	1.64	0.56	4.62	0.633
	YTE	2.49	3.10	1.29	0.35	12.25	
Gondoate (µg/mg)	Healthy	0.14	0.05	0.14	0.05	0.23	0.026
	YTE	0.41	0.67	0.19	0.09	2.64	
Docosanoate (µg/mg)	Healthy	0.18	0.07	0.17	0.07	0.34	0.002
	YTE	0.42	0.46	0.26	0.15	1.92	
Erucate (µg/mg)	Healthy	0.03	0.01	0.03	0.01	0.04	< 0.001
	YTE	0.10	0.12	0.06	0.02	0.51	
Nervonate (µg/mg)	Healthy	0.14	0.08	0.11	0.04	0.36	< 0.001
	YTE	0.46	0.45	0.28	0.17	1.71	
TM fatty acids (µg/mg)	Healthy	28.96	14.22	26.11	6.49	54.89	0.965
	YTE	32.45	23.28	23.31	10.86	95.62	

TM, total measured; YTE, Yorkshire Terrier enteropathy; SD, standard deviation.

.

In YTE dogs fecal concentrations of docosanoate (ρ = 0.59, *p* = 0.027), gondoate (ρ = 0.54, *p* = 0.047), palmitate (ρ = 0.58, *p* = 0.030), and arachidonate (ρ = 0.73, *p* = 0.003) correlated positively with WSAVA histopathology scores. Fecal concentrations of vaccinate (ρ = 0.59, *p* = 0.026) correlated positively with the abundance of *Faecalibacterium* in YTE dogs.

### Dysbiosis Index and fecal metabolomics in active YTE compared to YTE dogs in remission

#### Dysbiosis Index

In none of the YTE dogs with DI values >0 at the time of enrolment, the DI recovered after achieving clinical remission. In only 1 dog with decreased abundance of *Faecalibacterium* and a negative DI, *Faecalibacterium* abundance reached the reference interval after treatment. In none of the dogs the abundance of *Cl. hiranonis* recovered after achieving clinical remission. There was no statistically significant difference of the DI or the abundances of *Cl. hiranonis* and *Fusobacterium* between YTE dogs before and after treatment. (Table 5; Fig. 1).

**Table 5:** Summary statistics of the abundances of bacterial groups assessed for the DI in YTE before and after treatment.

		Reference	Mean	SD	Median	Minimum	Maximum	P value
Turicibacter Log DNA	YTE	4.6–8.1	6.53	1.34	6.46	4.33	8.41	0.722
	Remission		6.72	1.40	6.26	5.15	8.64	
E.coli Log DNA	YTE	0.9–8.0	5.84	2.55	6.81	1.39	9.18	0.742
	Remission		6.51	1.31	6.51	4.22	8.59	
Fusobacterium Log DNA	YTE	7.0–10.3	7.87	0.95	7.58	6.44	9.23	0.856
	Remission		7.80	0.92	7.71	6.52	9.39	
Faecalibacterium Log DNA	YTE	3.4–8.0	3.47	1.15	3.26	2.26	6.34	0.722
	Remission		3.76	1.62	3.10	2.10	7.42	
Blautia Log DNA	YTE	9.5–11.0	10.34	0.67	10.61	8.67	10.90	0.505
	Remission		9.94	0.71	10.00	8.64	10.80	
Cl. hiranonis Log DNA	YTE	5.1–7.1	3.89	2.65	4.23	0.10	7.22	0.126
	Remission		2.98	2.59	1.37	0.10	7.24	
Streptococcus Log DNA	YTE	1.9–8.0	5.65	2.26	5.07	2.28	8.91	0.424
	Remission		5.22	2.60	3.75	2.60	8.81	
Dysbiosis Index	YTE		1.32	5.45	2.11	-5.90	8.77	0.810
	Remission		2.29	4.57	3.46	-5.72	8.24	

DI, dysbiosis index; YTE, Yorkshire Terrier enteropathy; SD, standard deviation.

#### Fecal bile acid concentrations

The concentrations and the percentages of total fecal secondary bile acids as well as the ratio of secondary to primary bile acids decreased significantly in dogs with YTE after treatment. The concentrations and percentages of the secondary fecal bile acids lithocholic acid (LCA) and deoxycholic acid (DCA), as well as the percentage of the secondary bile acid chenodeoxycholic acid (CDCA) were significantly decreased in YTE dogs in clinical remission. Conversely, the percentage of total fecal primary bile acids and the percentage of the primary bile acid cholic acid (CA) increased significantly in YTE dogs in remission. (Table 6; Fig. 2).

**Table 6:** Summary statistics of bile acid concentrations in YTE dogs before and after treatment.

		Mean	SD	Median	Minimum	Maximum	P value
CA (ng/mg)	YTE	4231	5517	908	14	16,358	0.750
	Remission	7016	4944	7955	1677	15,127	
CDA (ng/mg)	YTE	606	899	285	21	3312	0.750
	Remission	1551	1066	1285	415	3796	
LCA (ng/mg)	YTE	628	707	269	28	1998	0.003
	Remission	231	324	85	21	1107	
DCA (ng/mg)	YTE	3993	4018	2122	222	9694	0.004
	Remission	1474	2276	453	243	7406	
UDCA (ng/mg)	YTE	122	130	53	31	460	0.590
	Remission	130	123	80	35	441	
TPBA (ng/mg)	YTE	4872	6372	1193	36	19,670	0.075
	Remission	8567	5956	9281	2130	18,924	
TSBA (ng/mg)	YTE	4743	4623	2636	313	11,347	0.013
	Remission	1835	2554	714	429	8548	
TBA (ng/mg)	YTE	9615	5089	9726	1616	20,783	0.765
	Remission	10,403	5269	10,567	3544	19,355	
SBA (%)	YTE	50	44	34	4	100	0.016
	Remission	22	26	10	2	73	
PBA (% )	YTE	50	44	66	0	96	0.016
	Remission	78	26	90	27	98	
CA (%)	YTE	42	38	55	0	89	0.021
	Remission	63	22	75	21	80	
CDA (%)	YTE	7	7	5	0	19	0.004
	Remission	15	5	16	6	21	
LCA (%)	YTE	7	7	4	0	21	0.003
	Remission	3	3	1	0	9	
DCA (%)	YTE	42	38	29	2	87	0.013
	Remission	17	24	5	2	63	
UDCA (%)	YTE	1	1	1	0	2	0.286
	Remission	2	2	1	0	6	
SBA to PBA	YTE	51	87	1	0	271	0.016
	Remission	1	1	0	0	3	
PBA to SBA	YTE	7	9	2	0	23	0.131
	Remission	14	15	9	0	44	

CA, cholic acid; CDA, chenodeoxycholic acid; LCA, lithocholic acid; DCA, deoxycholic acid; UDCA, ursodeoxycholic acid; TPBA, total primary bile acids; TSBA, total secondary bile acids; SBA to PBA; ratio of secondary to primary bile acids; PBA to SBA, ratio of primary to secondary bile acids; YTE, Yorkshire Terrier enteropathy; SD, standard deviation.

#### Fecal sterol concentrations

There was no statistically significant difference in any of the measured fecal sterol concentrations of the dogs during active disease or during clinical remission. (Table 7; Fig. 3).

**Table 7:** Summary statistics of fecal sterol concentrations in YTE dogs before and after treatment.

		Mean	SD	Median	Minimum	Maximum	P value
Coprostanol (µg/mg)	YTE	0.05	0.02	0.04	0.02	0.07	0.285
	Remission	0.04	0.02	0.04	0.03	0.10	
Cholesterol (µg/mg)	YTE	5.26	2.93	4.84	1.73	12.00	0.213
	Remission	4.80	3.08	4.39	1.85	13.26	
Cholestanol (µg/mg)	YTE	0.24	0.20	0.21	0.11	0.90	0.790
	Remission	0.18	0.08	0.15	0.11	0.38	
Brassicasterol (µg/mg)	YTE	0.05	0.02	0.05	0.04	0.10	0.248
	Remission	0.05	0.01	0.04	0.04	0.07	
Lathosterol (µg/mg)	YTE	0.04	0.04	0.03	0.01	0.18	0.091
	Remission	0.02	0.00	0.02	0.02	0.03	
Campesterol (µg/mg)	YTE	0.67	0.31	0.56	0.30	1.31	0.806
	Remission	0.71	0.28	0.69	0.42	1.39	
Stigmasterol (µg/mg)	YTE	0.17	0.09	0.18	0.06	0.35	0.313
	Remission	0.21	0.05	0.22	0.14	0.32	
Fusosterol (µg/mg)	YTE	0.05	0.05	0.03	0.02	0.21	0.213
	Remission	0.03	0.01	0.03	0.02	0.05	
Beta-sitosterol (µg/mg)	YTE	1.02	0.77	0.76	0.27	2.84	0.657
	Remission	0.76	0.30	0.64	0.44	1.42	
Sitostanol (µg/mg)	YTE	0.11	0.18	0.02	0.01	0.63	0.131
	Remission	0.04	0.03	0.02	0.02	0.10	
TM phytosterols	YTE	2.02	1.25	1.82	0.70	4.96	0.550
	Remission	1.76	0.62	1.56	1.07	2.88	
TM zoosterols	YTE	5.59	2.99	5.03	2.05	12.43	0.213
	Remission	5.04	3.15	4.65	2.01	13.70	
TM phyto- to zoosterols	YTE	0.47	0.41	0.37	0.06	1.48	0.050
	Remission	0.41	0.19	0.35	0.20	0.76	
TM sterols (µg/mg)	YTE	7.66	3.20	7.69	3.68	13.23	0.328
	Remission	6.85	3.56	6.65	3.27	16.56	
TM sterols to fatty acids	YTE	0.30	0.16	0.26	0.08	0.72	0.050
	Remission	0.43	0.29	0.35	0.22	1.26	

TM, total measured; TM Phyto- to Zoosterols; ratio of total measured phyto- to total measured zoosterols; TM Sterols to Fatty Acids; ratio of total measured sterols to total measured fatty acids; YTE, Yorkshire Terrier enteropathy; SD, standard deviation.

#### Fecal fatty acid concentrations

The fecal concentrations of the long-chain fatty acids docosanoate, gondoate, erucate, myristate, palmitate, linoleate, oleate, stearate, arachidonate, cis-vaccinate, as well as the total fecal fatty acids decreased significantly after treatment in YTE dogs (Table 8; Fig. 4).

**Table 8:** Summary statistics of fecal fatty acid concentrations in YTE dogs before and after treatment.

		Mean	SD	Median	Minimum	Maximum	P value
Myristate (µg/mg)	YTE	1.39	2.10	0.57	0.18	7.87	0.003
	Remission	0.16	0.06	0.15	0.06	0.27	
Palmitate (µg/mg)	YTE	6.63	4.34	4.79	2.49	16.97	0.008
	Remission	3.60	0.96	3.65	1.63	5.05	
Linoleate (µg/mg)	YTE	5.50	4.34	4.36	2.01	18.99	0.026
	Remission	3.69	1.71	3.09	1.71	6.95	
α-Linolenate (µg/mg)	YTE	0.94	0.84	0.73	0.26	3.51	0.929
	Remission	1.09	1.30	0.55	0.17	4.70	
Oleate (µg/mg)	YTE	5.64	4.32	4.30	1.80	15.64	0.006
	Remission	2.96	1.17	2.96	1.37	5.06	
Cis-vaccenate (µg/mg)	YTE	1.89	1.44	1.24	0.03	4.62	0.008
	Remission	0.57	0.18	0.55	0.25	0.81	
Stearate (µg/mg)	YTE	6.58	4.71	5.21	2.12	16.97	0.013
	Remission	3.49	1.07	3.84	1.46	5.11	
Arachidonate (µg/mg)	YTE	2.49	3.10	1.29	0.35	12.25	0.016
	Remission	1.42	0.77	1.22	0.53	3.02	
Gondoate (µg/mg)	YTE	0.41	0.67	0.19	0.09	2.64	0.021
	Remission	0.15	0.05	0.15	0.07	0.26	
Docosanoate (µg/mg)	YTE	0.42	0.46	0.26	0.15	1.92	0.004
	Remission	0.19	0.04	0.20	0.12	0.26	
Erucate (µg/mg)	YTE	0.10	0.12	0.06	0.02	0.51	0.016
	Remission	0.06	0.01	0.06	0.05	0.08	
Nervonate (µg/mg)	YTE	0.46	0.45	0.28	0.17	1.71	0.004
	Remission	0.20	0.04	0.18	0.14	0.26	
TM fatty acids (µg/mg)	YTE	32.45	23.28	23.31	10.86	95.62	0.004
	Remission	17.59	5.93	17.44	7.75	27.47	

TM, total measured; YTE, Yorkshire Terrier enteropathy; SD, standard deviation.

## Discussion

In the current study, the DI was used as a quantitative tool for assessment of intestinal dysbiosis and quantitative targeted metabolomics were used to identify changes in fecal metabolites in dogs with YTE compared to healthy Yorkshire Terriers, and to investigate the impact of treatment on both the microbiome and the fecal metabolome. This study revealed a high incidence of dysbiosis, an increased DI, and a lower abundance of *Fusobacterium* and *Cl. hiranonis* in dogs with YTE, compared to healthy Yorkshire Terriers. Furthermore, we identified changes in lipid and bile acid metabolism in symptomatic dogs with YTE. However, these changes were only partially resolved in dogs in clinical remission.

In accordance to previous studies in dogs with CE^10,17,18^, dogs with YTE had a higher DI compared to healthy control dogs, indicating gut dysbiosis. Furthermore, the DI increased with increased clinical severity of YTE. Gut dysbiosis refers to a disturbance of the intestinal microbiome, leading to functional deviations of the microbial transcriptome, proteome, or metabolome^7^. The DI is based on a mathematical algorithm, which was developed based on the results of molecular studies^19,20^, to calculate the degree of dysbiosis in dogs using fecal samples^8^. The fecal DI uses the quantification of the abundance of total bacteria and 7 bacterial groups (*Faecalibacterium spp., Turicibacter spp., Escherichia coli, Streptococcus spp., Blautia spp., Fusobacterium spp., and Cl. hiranonis*) that are commonly altered in dogs with CE. Based on the quantitative measurements by qPCR, reference intervals have been established for the fecal abundance of each bacterial group. A negative DI indicates that no shifts in the overall diversity of the intestinal microbiota have occurred. However, the abundance of individual bacterial groups outside their reference intervals suggests mild dysbiosis. A DI between 0 and 2 is currently defined as a mild to moderate shift in the intestinal microbiota and values > 2 are indicative of major shifts in the intestinal microbiota. Recent studies and a meta-analysis showing increased DI and alterations in these bacterial taxa in dogs with intestinal disease have confirmed the value of these parameters^10,17,18,21^. In our study dysbiosis in YTE was characterized by a decrease of the abundance of *Cl. hiranonis* and *Fusobacterium* compared to healthy control dogs*.* Furthermore, the abundance of *Faecalibacterium* was below the reference interval in a majority of YTE dogs. Various patterns of alterations in bacterial groups evaluated by the DI have been observed in dogs with CE in previous studies^10,17,18^ *Cl. hiranonis* has been shown to play an important role in the conversion of primary bile acids to secondary bile acids^22^ and decreases in the fecal abundance of *Cl. hiranonis* have been reported in dogs with CE^8,12^. *Fusobacterium* is associated with the production of short chain fatty acids from protein sources^23^. Decreased fecal abundances of *Faecalibacterium*, a short chain fatty acid producer, have also been reported in dogs with CE^10,13,17^. All three bacterial groups play a crucial role in lipid and bile acid metabolism and have a major influence on the gut metabolome. Metabolomic profiling allows for the analysis of small molecules in biological samples. It is a powerful tool to analyze metabolic changes associated with several pathological conditions. It is increasingly used for the discovery of etiological factors, disease signatures, and as a screening tool for different pathological conditions, including IBD^24^. Characterization of metabolic changes occurring in canine CE can increase our understanding of disease pathophysiology, which may improve diagnosis, treatment, and disease management.

In this study, dogs with YTE had significantly lower fecal concentrations of UDCA, a secondary bile acid, compared to healthy controls. This is consistent with recent studies revealing decreased secondary bile acid concentrations in fecal samples of dogs with CE^11,13,17^. A substantial proportion of humans with Crohn's disease and with diarrhea-predominant irritable bowel syndrome suffer from a condition known as bile acid or bile salt malabsorption, which is characterized by elevated total and primary fecal bile acids^25^. Furthermore, decreased expression of the active sodium-dependent bile acid transporter in the ileum of dogs with chronic enteropathy has been documented, indicating bile acid malabsorption in dogs with CE^18^. Interestingly, in our study the concentrations of total fecal bile acids correlated positively with increased severity of histological lesions in dogs with YTE. However, it seems that a disbalance rather than an excessive amount of colonic bile acids is more common in canine CE. Primary bile acids are synthesized by the liver and secreted into the intestine. In healthy animals, a small percentage of the intestinal bile acids are not reabsorbed and reach the colon, where colonic bacteria convert primary to secondary bile acids by deconjugation and dehydroxylation^26^. In our study we found a negative correlation of fecal primary bile acids with *Cl. hiranonis* abundance, while secondary bile acids were positively correlated with the abundance of this specific bacterial group. This supports the role of *Cl. hiranonis* as an important bile acid converter in dogs. YTE and dysbiosis may lead to bile acid dysmetabolism due to a decreased proportion of *Cl. hiranonis*, resulting in altered bile acid conversion.

The current study revealed perturbations in lipid metabolism in dogs with YTE. Lipids are a heterogeneous group of hydrophobic and amphiphilic molecules that are responsible for the maintenance of membrane structure and permeability and possess many regulatory functions in immunity and inflammation^27^. In this study, YTE dogs had increased fecal concentrations of the plant sterols campesterol and brassicasterol, while the plant sterol metabolite sitostanol concentrations were decreased. Decreased sitostanol concentrations have been previously described in dogs with IBD^28^. Sterols are a subtype of lipids occurring in cell membranes. While cholesterol is an essential component of animal cell membranes, sterols are the structural components of plant cell membranes. The intestinal absorption of cholesterol is significantly more efficient (30–60%) than plant sterol absorption (2–3%). However, plant sterols can reduce cholesterol absorption in the gastrointestinal tract and therefore have been studied mostly in relation to their hypocholesteremic effects^29^. Since plant sterols cannot be synthesized de novo by mammalian hosts, fecal concentrations resemble the end result of dietary ingestion and intestinal absorption. Therefore, malabsorption due to disturbed epithelial transport, a reduced intestinal absorptive area, and/or accelerated intestinal transit time accompanying gastrointestinal disease could all be reasons for increased fecal concentrations of plant sterols^30,31^. However, this would not explain decreased sitostanol concentrations identified in our study. Sterols do not pass the gastrointestinal tract unchanged, but unabsorbed sterols reach the colon where they are metabolized by intestinal bacteria^29,32^. Therefore, dysbiosis as seen in YTE dogs of our study could lead to disbalances in fecal sterols and their metabolites. We identified a positive correlation of fecal sitostanol concentrations and the fecal abundance of *Faecalibacterium*. The microbes responsible for sterol metabolism in dogs are unknown and the fecal abundance of *Faecalibacterium* was below the lower limit of the reference interval in the majority of dogs with YTE. This could indicate a role for *Faecalibacterium* in plant sterol metabolism or, alternatively, an influence of sterols on the abundance of *Faecalibacterium*. Altered fecal sterol concentrations could serve as markers for dysbiosis, and given the anti-inflammatory role of plant sterols in IBD animal models^33–35^, they could also play a role in the chronic inflammatory loop of YTE.

The current study revealed a significant increase of fecal long-chain free fatty acids in dogs with YTE, indicating lipid malabsorption or dysmetabolism. Elevated fecal fatty acid concentrations have been reported in human IBD patients, which is consistent with our results^36^. Increased fecal fatty acids could be a direct measure of fatty acid loss as a consequence of intestinal malabsorption. Decreased intestinal fatty acid absorptive capacity may be caused by disturbed epithelial transport, reduced intestinal absorptive area and/or accelerated intestinal transit time^30,31^. Moreover, YTE is often associated with dysfunctional intestinal lymphatics^14,15^, which may cause breed specific alterations in lipid absorption. Alternatively, altered fecal fatty acid profiles may indicate altered lipid cascades in inflammation and immune regulation. Fatty acids are known to play important roles in inflammation and immunity, trafficking, signal transduction, regulation of gene expression and autophagy. Furthermore, they are involved in the maintenance of the intestinal barrier and serve as antioxidants^27^. It has been shown that colonic inflammation in human IBD patients and IBD mouse models is associated with altered intestinal mucosal lipid profiles, which appear to be dependent on the degree of inflammation^37^. Similarly, we found that the concentration of individual fecal fatty acids increased with increased histological severity of intestinal lesions in YTE dogs. Fecal fatty acid concentrations could serve as markers for intestinal dysfunction or inflammation. Furthermore, they could be involved in a detrimental feedback loop where intestinal disease results in reduced fatty acid absorption and increased colonic long chain fatty acids may lead to diarrhea and epithelial cell damage^38–40^.

Normalization of DI or metabolomic profiles in dogs in clinical remission would be useful to monitor therapeutic responses in dogs with YTE and to identify patients with a disease relapse before the onset of clinical signs to allow early intervention. However, in the current study, clinical recovery of dogs with YTE did not correlate with the recovery of dysbiosis. A recent study demonstrated that although the DI in dogs with steroid-responsive CE normalized after three weeks of treatment, several bacterial taxa evaluated by qPCR and 16S rRNA sequencing were significantly different compared with healthy controls and did not normalize until one year after treatment response^41^. Another study in dogs with food responsive CE reported similar results with no recovery of the intestinal microbiome analysed by qPCR and 16S rRNA sequencing after a treatment period of 60 days^42^. Furthermore, a previous study evaluating the recovery of the DI in dogs with steroid responsive CE revealed that the DI was still increased after 3 months, despite clinical remission^17^. These data suggest that it takes several months to years for the gut microbiome to recover, likely due to changes at the intestinal mucosal level, and the timeframe chosen for reassessment in our study may have been insufficient to reach normobiosis. In addition, in treated YTE dogs of our study, bile acid dysbalance became even more pronounced with a decrease of various individual and total secondary bile acids and an increase in fecal primary bile acid concentrations. This is in contrast to results of a previous study in dogs with steroid responsive CE in which the fecal abundance of *Cl. hiranonis* significantly increased and bile acid metabolism was restored after two months of treatment^17^. Corticosteroids may improve bile acid metabolism by upregulation of the ileal active sodium-dependent bile acid transporter^43^, however this would not explain the recovery of *Cl. hiranonis* abundance*.* Furthermore, similar results were seen in dogs with CE reaching clinical remission after receiving a hydrolyzed protein diet^44^. Contrary to our results, the abundance of *Cl. hiranonis* increased and fecal bile acid concentrations normalized in dogs with food responsive disease after 2 weeks; which was in contrast to non-responders^44^. Therefore, it was suggested by the authors, that remission induced by a hydrolyzed protein diet is associated with improved dysbiosis, expansion of bile acid converters and, increasing concentrations of fecal secondary bile acids^44^. At least this does not appear to be the case in the dogs with YTE in our study, in which clinical recovery did not correlate with recovery of *Cl. hiranonis* abundance and bile acid dysbalance*.* In accordance to our results, feeding an extruded animal protein free diet to dogs with CE and healthy controls did not have an influence on the abundance of *Cl. hiranonis*^42^ and feeding a hydrolyzed diet in healthy dogs did not have an impact on *Cl. hiranonis* abundance or fecal bile acid concentrations^45^. While it is believed that dysbiosis represents the sequel rather than the cause of CE, we cannot exclude that gut dysbiosis and dysregulated intestinal metabolism is a predisposing factor for YTE and might persist in these dogs.

Similarly to the fecal DI and bile acid concentrations, despite clinical improvement fecal sterol concentrations did not normalize in YTE dogs. This might be expected if dysbiosis is responsible for an altered sterol pattern.

In contrast, long-chain fatty acids were recovered from the feces of dogs in clinical remission. This might indicate improved absorption by recovered lymphatics and intestinal mucosa and/or decreased intestinal inflammation. However, we did not reassess small intestinal biopsies in the treated IBD dogs of the present study, so we can only speculate on the intestinal histopathological appearance after clinical remission. Previous studies have shown that the histopathologic lesions in intestinal biopsies of dogs suffering from IBD did not change despite clinical improvement^46–49^. On the other hand, in a recent study feeding an omega-3 enriched diet increased the treatment response and resulted in marked suppression of intestinal inflammatory activity in IBD dogs^50^. Another explanation for our results could be that the diet itself had a beneficial influence on long-chain fatty acids by higher digestibility or a different fatty acid pattern.

Our study is not without limitations. The main limitation is the small number of dogs included, especially in the remission group. Another limitation is that dogs in the control group were older than dogs with YTE, which might have influenced our results. A previous study revealed that the canine intestinal gut microbiome showed a decreased microbial diversity and is likely to vary with increasing age^51^. However, the influence of age on the DI and the fecal metabolome in adult dogs have not yet been studied. Due to ethical concerns, we did not perform gastroduodenoscopy in dogs in clinical remission to assess recovery of intestinal inflammation and architectural changes. Furthermore, since we enrolled client-owned dogs, the dogs were not fed a standardized diet, which might have influenced the results of metabolite profiling. However, at the time of enrolment all dogs except one (receiving a BARF diet) were fed comparable commercially available diets. Additionally, in one dog in the treatment group prednisolone had to be added to the diet. This may have influenced our results, since prednisolone may have a direct effect on individual metabolites, and dietary non responders may represent a more severe disease phenotype. Other conditions causing intestinal inflammation or epithelial injury may share the observed alterations. To ensure clinical relevance, it might be necessary to include Yorkshire Terriers with other gastrointestinal disorders in the future.

In summary, our study showed changes in the fecal microbiome and the fecal metabolic profile in Yorkshire Terriers with YTE. Notably, a dysbalance in bile acid metabolism, sterols, and fatty acids was observed in YTE patients. Considering the important functions of these compounds in inflammation, further studies are needed to shed more light on the role of these metabolites in dogs with YTE. Recognizing and understanding breed specific differences provides a great opportunity to optimize our treatment approach in dogs with IBD.

## Materials and methods

The study was approved by the Ethics Committee of the University of Veterinary Medicine Vienna and the Austrian Federal Ministry of Science and Research (BMWF-68.205/0150-V/3b/2018). Before being enrolled into the study owners of each dog signed a written informed consent form.

### Cases and control dogs

Fourteen client-owned Yorkshire Terriers with a history of chronic (≥ 3-week duration) or intermittent gastrointestinal signs (vomiting, diarrhea, anorexia, and/or weight loss) or pleural or abdominal effusion presented to the Small Animal Internal Medicine Clinic of the University of Veterinary Medicine Vienna, Austria, were included in the study. The dogs were prospectively enrolled between November 2018 and March 2021. The diagnosis of YTE was based on the results of physical examination, haematological and biochemical parameters, urine analysis, abdominal ultrasonography, fecal parasitological examinations and gastroduodenoscopy results. For the exclusion of systemic, infectious, endocrine, and neoplastic causes for the presenting signs a complete blood count, serum biochemical analysis, the measurement of serum total bile acids, basal cortisol concentrations, cTLI (canine trypsin-like-immunoreactivity) and cPLI (canine pancreatic lipase immunoreactivity as measured by Spec cPL) and an ACTH-stimulation test (if the basal cortisol < 2 µg/dl) were performed. Additionally analysis of fecal samples by flotation and Giardia antigen test and abdominal ultrasonography were performed. Furthermore, for the exclusion of renal protein loss urinalysis including evaluation of the urine sediment and urine protein creatinine ratio (UPC) was performed. To confirm intestinal mucosal inflammation all YTE cases underwent gastroduodenoscopy.

The control group consisted of 26 adult Yorkshire Terriers without signs of gastrointestinal disease (n = 26). The dogs were prospectively enrolled in the study over the same time period and were healthy as determined by history, physical examination, blood count, chemical profile, fecal and urinalysis, and abdominal ultrasonography. Dogs with antibiotic or glucocorticoid pretreatment within the last 2 weeks or dogs that had received a specific diet designed for dogs with gastrointestinal disease within the last 2 months were excluded from the study.

Clinical scores were collected from all dogs using the canine chronic enteropathy activity index (CCECAI)^46^ at the time of study entry and additionally for YTE dogs at the control appointments. The initial diagnostic workup was performed by one board-certified internist (A.I.G.), who also performed the control visits. Endoscopic duodenal activity scores were calculated based on the presence (1 point each) or absence (0 points each) of friability, granularity, erosions, and lymphatic dilatation^52^.

Intestinal biopsies of the YTE dogs were graded by one blinded board-certified pathologist (B.R.), according to the World Small Animal Veterinary Association (WSAVA) International Gastrointestinal Standardization Group guidelines^2^.

All dogs with YTE were part of a treatment trial consisting of the initial feeding of either a hydrolyzed diet (Hill's Prescription Diet z/d Canine) or a low fat diet (Hill's Prescription Diet i/d Low Fat Canine). The diet was randomly assigned by random number generation and the owners and the specialist performing the work up and control appointments were blinded to the diets. The dogs were re-evaluated every 14 days. If the treatment was not successful (CCECAI score > 3), the dogs were switched to the other diet and rechecked after another 14 days. A weaning period was not considered for ethical reasons. The feeding of canned or dry food was at the discretion of the owner to increase the acceptability of the diet. The amount of feed was according to the manufacturer's instructions based on body weight. In case of non-response to dietary treatment alone (CCECAI > 3), additional administration of prednisolone (1 mg/kg q12h PO) was planned. In case of further non-response after another 14 days, cyclosporine (5 mg/kg q24h PO) would have been added as a second immunosuppressant. After achieving clinical remission (defined by a decrease in CCECAI scores to ≤ 3), at the earliest 70 days after treatment initiation, YTE dogs were re-examined, and fecal samples were again collected for DI and metabolomic analysis.

### Sample collection and storage

Fecal samples were collected from dogs with YTE at the time of diagnosis and after reaching clinical remission. The healthy control dogs were sampled once.

Fecal samples were collected from three consecutive defecations and frozen immediately. Fecal samples were stored frozen at − 80 °C and shipped on dry ice to the Gastrointestinal Laboratory, Department of Small Animal Clinical Sciences, Texas A&M University, College Station, TX, for analysis.

### Fecal Dysbiosis Index

For the calculation of the DI, quantitative PCR assays were performed for total bacteria, *Faecalibacterium, Turicibacter, Escherichia coli, Streptococcus, Blautia, Fusobacterium,* and *Clostridium hiranonis* as previously described^8,13^. The results were expressed as log DNA abundance (femtogram) for each particular bacterial group per 10 nanogram of total isolated DNA.

### Fecal metabolome analysis

A targeted metabolomic approach was used to measure fecal concentrations of selected unconjugated bile acids (i.e., cholic acid, chenodeoxycholic acid,lithocholic acid, deoxycholic acid, ursodeoxycholic acid), cholesterol and cholesterol intermediates (i.e., coprostanol, cholestanol, lathosterol), plant sterols (i.e., β-sitosterol, brassicasterol, campesterol, fucosterol, sitostanol, stigmasterol) and long chain fatty acids (i.e., palmitate, linoleate, α-linolenate, oleate, cis-vaccenate, stearate, arachidonate, gondoate, erucate, docosonoate, nervonate). A previously described protocol for the simultaneous quantitation of fatty acids, sterols and bile acids in human stool was adapted and modified^53^. An aliquot of 10–14 mg of a lyophilized fecal sample was weighted in a 7 mL glass centrifuge tube for further sample processing. The weight of each sample was recorded for the calculation of the final concentration. A mixture of 160 μL 1-butanol, 10 μL each d7-sitostanol, d6-cholesterol, d4-stearic acid, and d4-cholestane (2 mg/mL each), and 20 μL each of d4-cholic acid and d4-lithocholic acid (1 mg/mL each) was added to each sample. A 20 μL concentrated HCl was added to each tube and vortexed for at least 30 seconds, before the tube was incubated at 65 °C for 4 hours (ThermoScientific, REACTI-Therm III #TS-18824 Heating module). After 4-hour incubation, samples were briefly vortexed, then dried with heat on under nitrogen gas until visibly dry. A 200 μL of a commercial silylating mixture (Sylon HTP, a mixture of hexamethyldisilazane, chlorotrimethylsilane, and pyridine = 2:1:10 (v/v/v)) was added to each dried tube for 30-min incubation at 65 °C. After incubation, tubes were again vortexed briefly before drying under heat and nitrogen flow. A 200 μL of hexane was added to each tube and briefly vortexed. Tubes were then centrifuged at 18.0 g force (3000 rcf) at 5 °C for 10 min (Eppendorf Centrifuge 5810 R). 50 μL of supernatant was transferred to a glass vial insert for further analysis.

One microliter of the sample was injected by an autosampler (7693A, Agilent Technologies, Palo Alto, USA) to a gas chromatography (GC) system (8890 GC system, Agilent Technologies) coupled with a mass spectrometer (MS, 5977B GC/MSD, Agilent Technologies). The gas chromatographic conditions were as followed: a DB-1MS Ultra Inert column (Agilent, 30 m × 0.25 mm I.D. and 0.25 μm film thickness) was used; helium was used as the carrier gas at a constant flow rate of 1 mL/min; a 1 μL volume of sample was injected in a 20:1 split with a split liner (taper, low pressure drop, with glass wool); inlet temperature was at 250 °C; oven temperature was initially held at 150 °C for 1 min, ramped to 276 °C at 21 °°C/min, and held for 15 min with a post-run time of 3 minutes at 325 °C. The MS was run in an SIM mode (selected ion monitoring) for quantitative analysis, using ion fragments for quantitation and verification. The software (ChemStation, Agilent) was used to automatically integrate all peaks and calculate the concentrations of analytes (μg/mL) in the injected hexane solution. These data were exported and the recorded weight of lyophilized feces for each sample was used to calculate concentrations in μg or ng per mg of lyophilized feces.

Data for the assessment of bile acids are reported as total amounts in micrograms per milligram of lyophilized fecal content and as percent of total bile acids measured. Total primary bile acids comprise the sum of cholic acid and chenocholic acid, while total secondary bile acids comprise the sum of lithocholic acid, desoxycholic acid, and ursodeoxycholic acid. Total bile acids represent the sum of all bile acids measured by the assay. Furthermore, the ratio of primary to secondary bile acids and the ratio of secondary to primary bile acids were calculated.

Total measured phytosterols comprise the sum of β-sitosterol, brassicasterol, fucosterol, campesterol, sitostanol, and stigmasterol. Total measured zoosterols comprise the sum of cholesterol, coprostanol, cholestenol, and lathosterol. Total measured sterols comprise the sum of phyto- and zoosterols. Furthermore, the ratio of phyto- to zoosterols and the ratio of sterols to fatty acids were calculated. Total measured fatty acids comprise the sum of all fatty acids measured by the assay.

### Statistics

Age and gender between YTE dogs and healthy control dogs were compared by a Wilcoxon rank test. Gender between YTE dogs and healthy Yorkshire Terriers was compared by Fishers exact test. Data were tested for normal distribution by Shapiro Wilk tests. Data were compared between healthy and YTE dogs by use of a Students t-test in case of normal distribution or a Mann Witney U-test in case of non-normal distribution. Data of dogs before and after the treatment trial were compared by a paired T-test or a Wilcoxon signed rank test, dependent on normality testing. Spearman rank test was used to test for correlations between fecal bile acid, sterol, and fatty acid concentrations to clinical, endoscopic, histological scores, the DI, and the fecal abundance of bacterial strains. *P* < 0.05 were considered significant. Data analysis was performed by SPSS version 20 (SPSS GmbH Software).

### Ethical approval

This article does not contain any studies with human participants performed by any of the authors.

### Research involving animal rights

All applicable international, national, and/or institutional guidelines for the care and use of animals were followed. The study was approved by the Ethics Committee of the University of Veterinary Medicine Vienna and the Austrian Federal Ministry of Science and Research (BMWF-68.205/0150-V/3b/2018). The study was carried out with adherence to a high standard (best practice) of veterinary care. Before being enrolled into the study owners of each dog signed a written informed consent form. All methods were performed in accordance with the relevant guidelines and regulations. According to the editorial policy for scientific reports, the study was carried out in compliance with the ARRIVE guidelines.

## Data Availability

The datasets generated and/or analyzed during the current study are available from the corresponding author on reasonable request.

## References

[1] Craven M, Simpson JW, Ridyard AE, Chandler ML (2004). Canine inflammatory bowel disease: Retrospective analysis of diagnosis and outcome in 80 cases (1995–2002). J. Small Anim. Pract..

[2] Washabau RJ (2010). Endoscopic, biopsy, and histopathologic guidelines for the evaluation of gastrointestinal inflammation in companion animals. J. Vet. Intern. Med..

[3] Sartor RB (2006). Mechanisms of disease: pathogenesis of Crohn's disease and ulcerative colitis. Nat. Clin. Pract. Gastroenterol. Hepatol..

[4] Xavier RJ, Podolsky DK (2007). Unravelling the pathogenesis of inflammatory bowel disease. Nature..

[5] Fritz JH, Le Bourhis L, Magalhaes JG, Philpott DJ (2008). Innate immune recognition at the epithelial barrier drives adaptive immunity: APCs take the back seat. Trends Immunol..

[6] Brant SR (2013). Promises, delivery, and challenges of inflammatory bowel disease risk gene discovery. Clin. Gastroenterol. Hepatol..

[7] Pilla R, Suchodolski JS (2020). The role of the canine gut microbiome and metabolome in health and gastrointestinal disease. Front. Vet. Sci..

[8] AlShawaqfeh M (2017). A dysbiosis index to assess microbial changes in fecal samples of dogs with chronic inflammatory enteropathy. FEMS Microbiol. Ecol..

[9] Patti GJ, Yanes O, Siuzdak G (2012). Innovation: Metabolomics the apogee of the omics trilogy. Nat. Rev. Mol. Cell Biol..

[10] Minamoto Y (2019). Fecal short-chain fatty acid concentrations and dysbiosis in dogs with chronic enteropathy. J. Vet. Intern. Med..

[11] Honneffer JB (2015). Untargeted metabolomics reveals disruption within bile acid, cholesterol, and tryptophan metabolic pathways in dogs with idiopathic inflammatory bowel disease. Gasteroentrology..

[12] Xu J (2016). Does canine inflammatory bowel disease influence gut microbial profile and host metabolism?. Vet. Res..

[13] Blake AB (2019). Altered microbiota, fecal lactate, and fecal bile acids in dogs with gastrointestinal disease. PLoS ONE..

[14] Kimmel SE, Waddell LS, Michel KE (2000). Hypomagnesemia and hypocalcemia associated with protein-losing enteropathy in Yorkshire terriers: five cases (1992–1998). J. Am. Vet. Med. Assoc..

[15] Simmerson SM (2014). Clinical features, intestinal histopathology, and outcome in protein-losing enteropathy in Yorkshire Terrier dogs. J. Vet. Intern. Med..

[16] Dijkstra M, Kraus JS, Bosje JT, Den Hertog E (2010). Protein-losing enteropathy in Rottweilers. Tijdschr. Diergeneeskd..

[17] Guard BC (2019). Longitudinal assessment of microbial dysbiosis, fecal unconjugated bile acid concentrations, and disease activity in dogs with steroid-responsive chronic inflammatory enteropathy. J. Vet. Intern. Med..

[18] Giaretta PR (2018). Comparison of intestinal expression of the apical sodium-dependent bile acid transporter between dogs with and without chronic inflammatory enteropathy. J. Vet. Intern. Med..

[19] Vazquez-Baeza Y, Hyde ER, Suchodolski JS, Knight R (2016). Dog and human inflammatory bowel disease rely on overlapping yet distinct dysbiosis networks. Nat. Microbiol..

[20] Honneffer JB (2014). Microbiota alterations in acute and chronic gastrointestinal inflammation of cats and dogs. World J. Gastroenterol..

[21] Félix AP, Souza CMM, de Oliveira SG (2022). Biomarkers of gastrointestinal functionality in dogs: A systematic review and meta-analysis. Anim. Feed. Sci. Technol..

[22] Kitahara M, Takamine F, Imamura T, Benno Y (2001). Clostridium hiranonis sp. nov., a human intestinal bacterium with bile acid 7alpha-dehydroxylating activity. Int. J. Syst. Evol. Microbiol..

[23] Potrykus J, White RL, Bearne SL (2008). Proteomic investigation of amino acid catabolism in the indigenous gut anaerobe Fusobacterium varium. Proteomics..

[24] Smirnov KS (2016). Challenges of metabolomics in human gut microbiota research. Int. J. Med. Microbiol..

[25] Walters JR, Pattni SS (2010). Managing bile acid diarrhea. Ther. Adv. Gastroenterol..

[26] Hofmann AF (1999). The continuing importance of bile acids in liver and intestinal disease. Arch. Intern. Med..

[27] Li M, Fan P, Wang Y (2015). Lipidomics in health and diseases—Beyond the analysis of lipids. Glycomics Lipidomics.

[28] Honneffer J. B. (2017). Microbiota and metabolomic changes across various canine gastrointestinal diseases [PhD thesis, Texas A&M University). Retrieved from https://scholars.library.tamu.edu/vivo/display/n98a05e2f

[29] Cuevas-Tena M, Alegría A, Lagarda MJ (2018). Relationship between dietary sterols and gut microbiota: a review. Eur. J. Lipid Sci. Technol..

[30] Montoro-Huguet MA, Belloc B, Domínguez-Cajal M (2021). Small and large intestine (I): Malabsorption of nutrients. Nutrients..

[31] Heimerl S (2006). Alterations in intestinal fatty acid metabolism in inflammatory bowel disease. Biochim. Biophys. Acta..

[32] Cuevas-Tena M (2018). Plant sterols and human gut microbiota relationship: An in vitro colonic fermentation study. J. Funct. Foods.

[33] Aldini R (2014). Antiinflammatory effect of phytosterols in experimental murine colitis model: Prevention, induction, remission study. PLoS One..

[34] Mencarelli A, Renga Palladino G, Distrutti E, Fiorucci S (2009). The plant sterol guggulsterone attenuates inflammation and immune dysfunction in murine models of inflammatory bowel disease. Biochem. Pharmacol..

[35] te Velde AA (2015). Effects of dietary plant sterols and stanol esters with low- and high-fat diets in chronic and acute models for experimental colitis. Nutrients..

[36] Jansson J (2009). Metabolomics reveals metabolic biomarkers of Crohn’s disease. PLoS One..

[37] Fernández-Bañares F (1997). Changes in mucosal fatty acid profile in inflammatory bowel disease and in experimental colitis: A common response to bowel inflammation. Clin. Nutr..

[38] Kim YS, Spritz N (1968). Hydroxy acid secretion in steatorrhea of pancreatic and non-pancreatic origin. N. Engl. J. Med..

[39] Soong CS, Thompson JB, Poley JR, Hess DR (1972). Hydroxy fatty acids in human diarrhea. Gastroenterology..

[40] Ramakrishna BS, Mathan M, Mathan VI (1994). Alteration of colonic absorption by long-chain unsaturated fatty acids—Influence of hydroxylation and degree of unsaturation. Scand. J. Gastroenterol..

[41] Pilla R (2021). Long-term recovery of the fecal microbiome and metabolome of dogs with steroid-responsive enteropathy. Animals (Basel).

[42] Bresciani F (2018). Effect of an extruded animal protein-free diet on fecal microbiota of dogs with food-responsive enteropathy. J. Vet. Intern. Med..

[43] Nowicki M (1997). Glucocorticoids upregulate taurocholate transport by ileal brush-border membrane. Am. J. Physiol. Gastrointest. Liver Physiol..

[44] Wang S (2019). Diet-induced remission in chronic enteropathy is associated with altered microbial community structure and synthesis of secondary bile acids. Microbiome..

[45] Pilla R (2020). Effects of metronidazole on the fecal microbiome and metabolome in healthy dogs. J. Vet. Intern. Med..

[46] Schreiner NMS, Gaschen F, Grone A, Sauter SN, Allenspach K (2008). Clinical signs, histology, and CD3-positive cells before and after treatment of dogs with chronic enteropathies. J. Vet. Intern. Med..

[47] Garcia-Sancho M, Rodriguez-Franco F, Sainz A, Mancho C, Rodriguez A (2007). Evaluation of clinical, macroscopic, and histopathologic response to treatment in non-hypoproteinemic dogs with lymphocytic-plasmacytic enteritis. J. Vet. Intern. Med..

[48] Allenspach K, Wieland B, Gröne A, Gaschen F (2007). Chronic enteropathies in dogs: Evaluation of risk factors for negative outcome. J. Vet. Intern. Med..

[49] Burgener IA (2008). Upregulation of toll-like receptors in chronic enteropathies in dogs. J. Vet. Intern. Med..

[50] Ontsouka EC, Burgener IA, Luckschander-Zeller N, Blum JW, Albrecht C (2012). Fish-meal diet enriched with omega-3 PUFA and treatment of canine chronic enteropathies. Eur. J. Lipid. Sci. Tech..

[51] Mizukami K (2019). Age-related analysis of the gut microbiome in a purebred dog colony. FEMS Microbiol. Lett..

[52] Slovak JE (2015). Development and validation of an endoscopic activity score for canine inflammatory bowel disease. Vet. J..

[53] Batta AK (2002). Simultaneous quantitation of fatty acids, sterols and bile acids in human stool by capillary gas-liquid chromatography. J. Chromatogr. B Anal. Technol. Biomed. Life Sci..

